# Diagnostic value of a novel automated simultaneous amplification and testing method in pulmonary tuberculosis and extrapulmonary tuberculosis

**DOI:** 10.3389/fmicb.2025.1590635

**Published:** 2025-05-14

**Authors:** Xichao Ou, Peilei Hu, Xundi Bao, Zhou Liu, Chong Teng, Jingwei Guo, Dongfang Xu, Yue Li, Bing Zhao, Ruida Xing, Hui Xia, Ling Ma, Yang Zhou, Yang Zheng, Yuanyuan Song, Shengfen Wang, Yanlin Zhao, Yunhong Tan, Huiwen Zheng

**Affiliations:** ^1^National Key Laboratory of Intelligent Tracking and Forecasting for Infectious Diseases, National Center for Tuberculosis Control and Prevention, Chinese Centre for Disease Control and Prevention, Beijing, China; ^2^Department of Clinical Laboratory, Hunan Chest Hospital, Changsha, China; ^3^Department of Clinical Laboratory, Anhui Chest Hospital, Hefei, China; ^4^Department of Tuberculosis, Beijing Dongcheng District Center for Disease Control and Prevention, Beijing, China; ^5^Institute of Tuberculosis Prevention and Control, Gansu Provincial Center for Disease Control and Prevention, Lanzhou, China; ^6^Beijing Key Laboratory of Pediatric Respiratory Infection Diseases, Key Laboratory of Major Diseases in Children, Ministry of Education, National Clinical Research Center for Respiratory Diseases, Laboratory of Respiratory Diseases, Beijing Pediatric Research Institute, Beijing Children’s Hospital, Capital Medical University, National Center for Children’s Health, Beijing, China

**Keywords:** pulmonary tuberculosis, extrapulmonary tuberculosis, AutoSAT, diagnosis, Xpert MTB/RIF

## Abstract

**Objective:**

To evaluate the clinical diagnostic ability of automated simultaneous amplification and testing (AutoSAT) method in suspected pulmonary tuberculosis (PTB) and extrapulmonary tuberculosis (EPTB) by comparing with Xpert MTB/RIF (Xpert) method against the composite reference standard.

**Method:**

Patients with suspected PTB or EPTB were enrolled consecutively from two provincial tuberculosis designated hospitals between August 2022 and December 2023. Clinical specimens were collected for routine clinical tests and AutoSAT.

**Results:**

The 297 patients including 90 PTB suspects and 207 EPTB suspects were eligible for final analysis. Among the 63 confirmed PTB, the sensitivity of Xpert was significantly higher than that of AutoSAT (30.16% vs. 28.57%, *p* < 0.001). Of the 18 bacteriologically confirmed PTB, equal sensitivity (94.44%) was observed between Xpert and AutoSAT. The higher specificity of AutoSAT than Xpert (100% vs. 96.30%, *p =* 0.803) was observed among PTB. The higher sensitivity of AutoSAT than Xpert was observed among confirmed EPTB (22.07% vs. 18.62%, *p* < 0.001) and bacteriologically confirmed EPTB patients (92.31% vs. 84.62%, *p* = 0.53), but the specificity (100%) was equal. And AutoSAT yielded higher sensitivity on pleural fluid (14.91% vs. 9.65%, *p* = 0.276). The same specificities (100%) were observed on different types of specimens.

**Conclusion:**

AutoSAT is an accurate, sensitive and rapid method for the detection of Mycobacterium tuberculosis in both PTB and EPTB patients. AutoSAT is highly effective in bacteriologically confirmed PTB, and outperforms Xpert in paucibacillary EPTB patients, especially for pleural fluid specimen detection.

## Introduction

With an estimated 1.25 million deaths globally, tuberculosis (TB) returns to the top single cause of death in 2023 (World Health Organization, 2024). Although substantial progress in controlling tuberculosis has been made during the past three decades, China still ranks the third largest TB burden in the world, which is far from achieving the End TB Strategy targets by 2030 (World Health Organization, 2024). Rapid and accurate detection technology is critical in assisting the early diagnosis of TB and preventing the transmission of TB. Several nucleic acid amplification tests (NAATs) endorsed by World Health Organization (WHO) have been proven to be highly sensitive and specific to diagnosis of *Mycobacterium tuberculosis* complex (MTBC) in the past decade (World Health Organization, 2021; World Health Organization, 2013; World Health Organization, 2020; Chakravorty et al., 2017; Nathavitharana et al., 2017). However, the expensive specialized detection equipment, and the absence of the necessary infrastructure and resources restricted the widespread application in clinical laboratories in China.

The automated simultaneous amplification and testing (AutoSAT) method (Rendu Biotechnology Co. Ltd., Shanghai, China), based on a previously described SAT method for *M. tuberculosis* detection (Cui et al., 2012; Yan et al., 2016), is a novel molecular technique that integrates nucleic acid extraction, amplification, and detection, which was intended for point-of-care testing for TB. In the AutoSAT assay, the target 16S rRNA was reverse transcribed into cDNA by Moloney murine leukemia virus reverse transcriptase. Subsequently, multiple RNA copies were generated from the cDNA template using T7 RNA polymerase. These RNA products underwent another round of reverse transcription, and the accumulated cDNAs were detected by fluorescence-labeled specific probes. It simplified the cumbersome manual operations, with the first batch of 6 results delivered in 90 min, followed by 6 results every 10 min. Therefore, it has high throughput with 500 tests performed per day. Previous studies have reported high levels of sensitivity and specificity for SAT in the diagnosis of pulmonary tuberculosis (PTB) and extrapulmonary tuberculosis (EPTB), and it is capable of detecting live bacteria by targeting RNA (Cui et al., 2012; Li et al., 2020; Yuan et al., 2014). Besides, the technique is also cost effective by performed on conventional real-time PCR instrument, and with outstanding reproducibility. However, no data on the diagnostic accuracy of AutoSAT for PTB and EPTB are available, which are necessity to be evaluated prior to clinical application in China. In this study, we aimed to evaluate the clinical diagnostic ability of AutoSAT in suspected PTB and EPTB by comparing with Xpert MTB/RIF (Xpert) method against the composite reference standard (CRS).

## Materials and methods

### Study design and patients

Patients were enrolled consecutively from Anhui Chest Hospital and Hunan Chest Hospital between August 2022 and December 2023. Inclusion criteria were: suspected PTB or EPTB; completion of interferon-gamma release assay (IGRA) at initial visit; provision of one adequate qualified specimen; signed informed consent. Exclusion criteria were: anti-tuberculosis treatment exceeded more than 2 weeks within the past 1 month; unqualified samples; patient refusal to participate. The CRS diagnosis was confirmed by a combination of clinical, histopathological, laboratory, and radiological evidence. The bacteriologically confirmed TB was defined as cases with the positive results of smear microscopy or culture. The final clinical diagnosis of PTB and EPTB was made according to the definition previously published (World Health Organization, 2020). Non-TB cases were defined as a definitive diagnosis of another disease. Quality control of clinical diagnosis results were performed by clinical experts, who will review the relevant cases diagnosed in the project areas to confirm the final diagnosis results. The clinical information, including gender, age, clinical diagnosis, and examination results, was collected retrospectively. The study was approved by the Institutional Review Board of Anhui Chest Hospital (K2022-022) and Hunan Chest Hospital (LS2022082301).

### Laboratory study

Sputum was collected from suspected TB patients, and pleural fluid, urine, pus, pericardial effusion, or cerebrospinal fluid were collected from suspected EPTB patients. All specimens were sent to the laboratory for the above routine clinical tests. Sputum and pus were subjected to Ziehl-Neelsen staining directly, and the other extrapulmonary specimens were centrifuged at 3000 × g for 20 min before smear preparation. Followed by digested in N-acetyl-L-cysteine NaOH-Na citrate (1.5% final concentration) and neutralized with phosphate buffer (PBS, 0.067 mol/L, pH = 7.4), specimens were inoculated in Bactec MGIT 960 system for 6 weeks for mycobacterial culture. Positive cultures were subjected to para-nitrobenzoic acid/thiophene-2-carboxylic acid hydrazide (PNB/TCH) medium to distinguish MTB from NTM. For Xpert MTB/RIF assay, 1 mL processed specimen was mixed with 2 mL sample reagent, incubating at room temperature for 10 min, followed by transferred into cartridges and loaded into the GeneXpert instrument. For interferon-gamma release assay (IGRA) assay, 1 mL of whole blood was collected into each of the three separate test tubes, incubating for 16–24 h at 37°C, followed by centrifuged and collected the supernatant to assess the concentration of IFN-*γ* (IU/mL) via ELISA. All tests were performed at the TB reference laboratory in Anhui Chest Hospital and Hunan Chest Hospital, following the manufacturer’s instructions and adhering to the Chinese Laboratory Science Procedure of Diagnostic Bacteriology in Tuberculosis guidelines, with quality control routinely performed.

### AutoSAT

**Figure 1:**
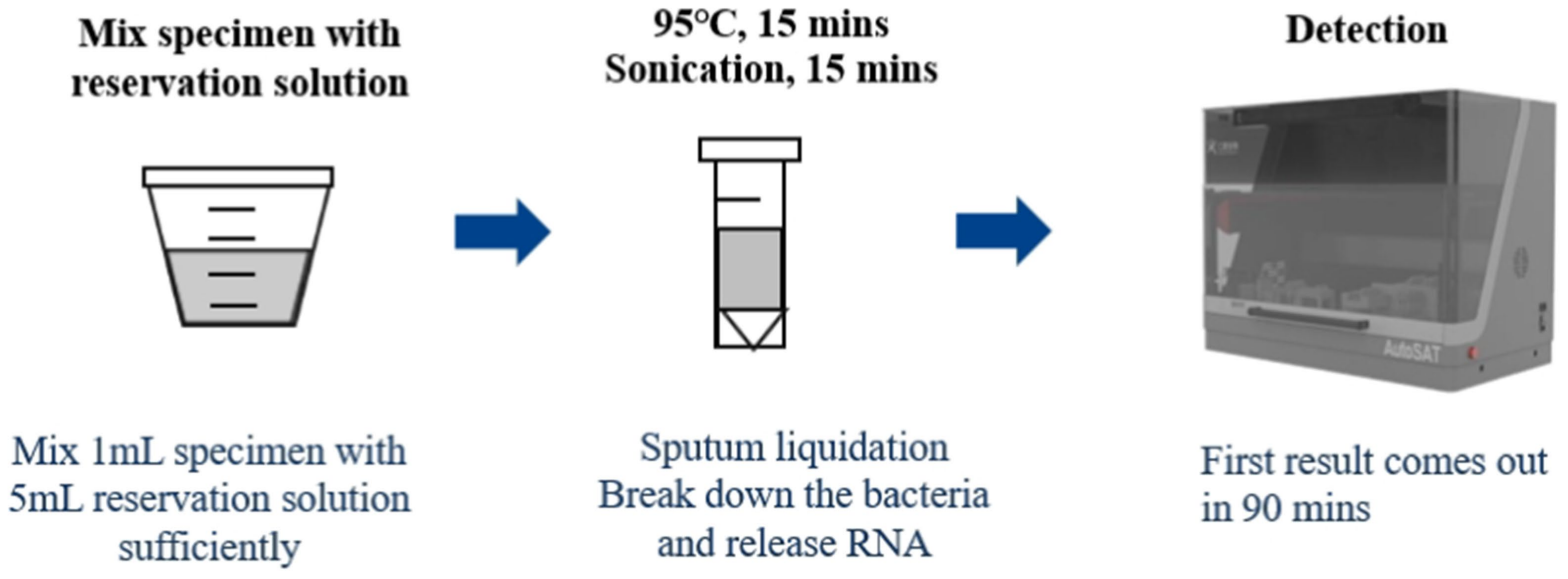
Workflow for AutoSAT assay.

One milliliter of resuspended sediment after centrifuged, 2 μL positive control and 1 mL saline, and 2 μL negative control and 1 mL saline were mixed with 1.5 mL GY01, respectively, followed by heating at 95°C for 15 min, then sonicated for 15 min at room temperature. Finally, the processed specimen, positive and negative controls were loaded into the AutoSAT system for further extraction, amplification, and detection (Figure 1). Fluorescence fluorescence signal was acquired after each PCR amplification cycle. For quality control, the following criteria were applied: for negative control, no S type amplification curve detected in the PCR batch; for positive control, clear S type amplification curve with a cycle threshold (CT) value <30; for DNA sample, CT value <45 in the internal standard channel. Subject to the quality control results, a CT value ≤35 was interpreted as positive for tuberculosis. Samples with CT values in the indeterminate range (35 < CT < 40) underwent repeat testing, with ultimately classified as positive if CT < 40 or negative if CT ≥ 40. The sample was considered TB-negative if the CT value>40. All personnel involved in the project have relevant professional backgrounds and have received unified training for the project.

### Statistical analysis

The diagnostic performance of AutoSAT and Xpert MTB/RIF was compared against the gold standard of bacteriologically diagnosis and composite reference standard, respectively. The sensitivity, specificity, positive predictive value (PPV), negative predictive value (NPV), and diagnostic accuracy of AutoSAT were compared with the Xpert MTB/RIF method for detecting *M. tuberculosis*. Concordance of the AutoSAT and Xpert result was performed using the Cohen’s Kappa test. Kappa ≥ 0.85 indicates excellent agreement between test results; 0.6 ≤ Kappa < 0.85 suggests good agreement; 0.45 ≤ Kappa < 0.6 indicates moderate agreement; Kappa < 0.45 reflects poor agreement. All analyses were done with SPSS 25.0 (SPSS Inc., USA), and *p* < 0.05 was regarded as statistically significant.

## Results

### Study population

**Figure 2:**
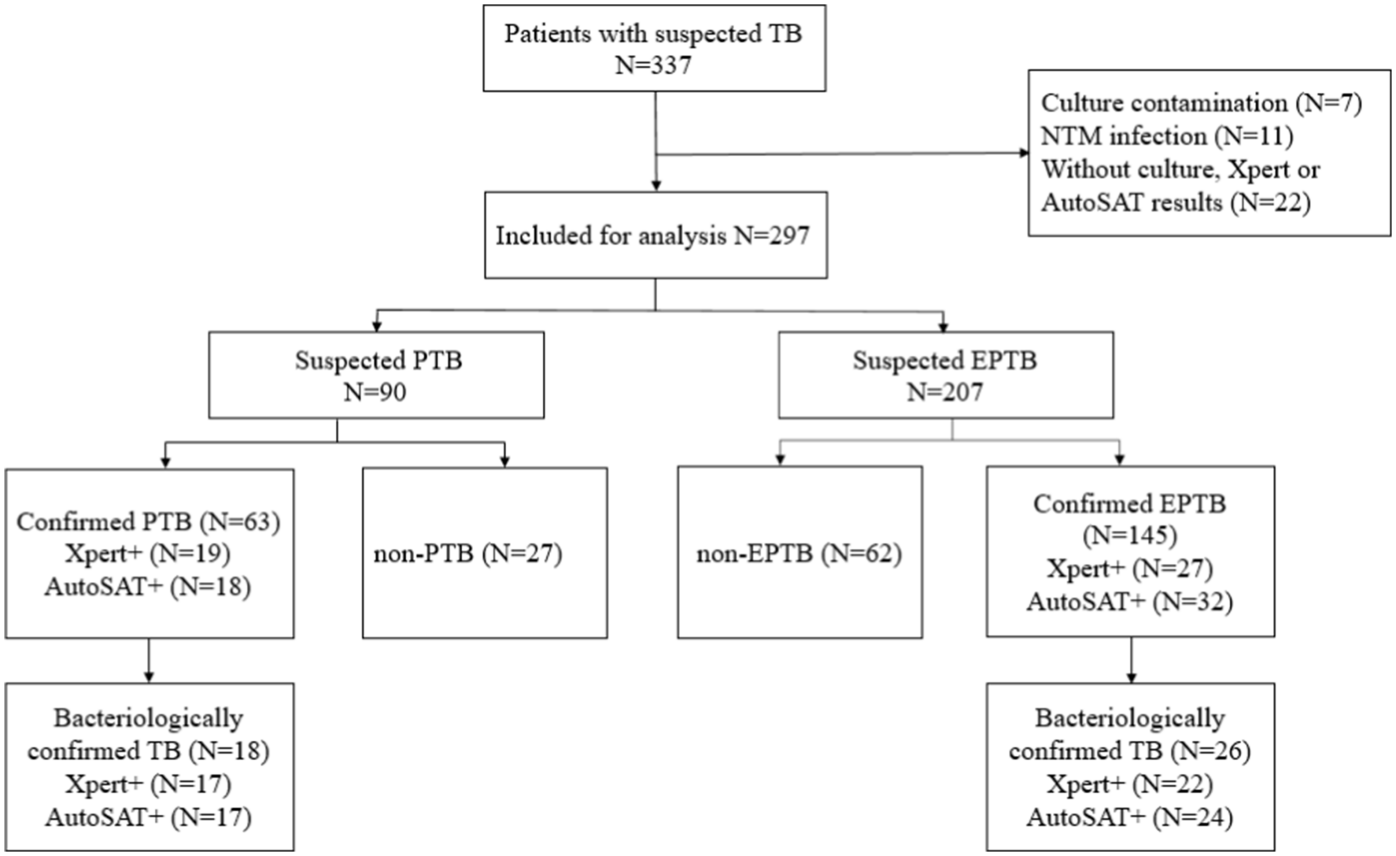
Patients enrollment and categories for patients with suspected TB.

A total of 337 suspected TB patients were initially enrolled. After excluding 7 cases with culture contamination, 11 patients with NTM infection, 22 individuals without culture or Xpert or AutoSAT results, a total of 297 patients including 90 PTB suspects and 207 EPTB suspects were eligible for final analysis (Figure 2). Of the 90 suspected PTB patients, 60 (66.67%, 60/90) were male, and 41.11% (37/90) patients were in range 45–64 years. Moreover, 63 (70.00%, 63/90) patients were confirmed PTB with 28.57% (18/63) bacteriologically positive. Among 207 suspected EPTB patients, 147 (71.01%, 147/207) were male, and 37.68% (78/207) patients were ≥65 years. The 145 (70.05%, 145/207) patients were confirmed EPTB with 17.93% (26/145) bacteriologically positive. The predominant types of extrapulmonary specimens were pleural fluid (77.29%, 160/207) and cerebrospinal fluid (7.73%, 16/207) (Table 1).

**Table 1:** Demographic characteristics of patients included for analysis and specimen types.

		Suspected PTB *N* = 90 (%)	Suspected EPTB *N* = 207 (%)	Total *N* = 297 (%)
Sex	Male	60 (66.67%)	147 (71.01%)	207 (69.70%)
	Female	30 (33.33%)	60 (28.99%)	90 (30.30%)
Age	<25	10 (11.11%)	19 (9.18%)	29 (9.76%)
	25–44	20 (22.22%)	34 (16.43%)	54 (18.18%)
	45–64	37 (41.11%)	76 (36.71%)	113 (38.05%)
	≥65	23 (25.56%)	78 (37.68%)	101 (34.01%)
IGRA	Positive	15 (16.67%)	70 (33.82%)	85 (28.62%)
	Negative	72 (80.00%)	132 (63.77%)	204 (68.69%)
	NA	3 (3.33%)	5 (2.42%)	8 (2.69%)
Smear	Positive	8 (8.89%)	2 (0.97%)	10 (3.37%)
	Negative	82 (91.11%)	186 (89.86%)	268 (90.24%)
	NA	-	19 (9.18%)	19 (6.40%)
Culture	Positive	18 (20.00%)	26 (12.56%)	44 (14.81%)
	Negative	72 (80.00%)	181 (87.44%)	253 (85.19%)
Xpert MTB/RIF	Positive	20 (22.22%)	27 (13.04%)	47 (15.82%)
	Negative	70 (77.78%)	180 (86.96%)	250 (84.18%)
AutoSAT	Positive	18 (20.00%)	32 (15.46%)	50 (16.84%)
	Negative	72 (80.00%)	175 (84.54%)	247 (83.16%)
Final diagnosis	Positive	63 (70.00%)	145 (70.05%)	208 (70.03%)
	Negative	27 (30.00%)	62 (29.95%)	89 (29.97%)
Specimen	Sputum	90 (100%)	–	90 (30.30%)
	Pleural fluid	–	160 (77.29%)	160 (53.87%)
	Pus	–	13 (6.28%)	13 (4.38%)
	Cerebrospinal fluid	–	16 (7.73%)	16 (5.39%)
	Urine	–	6 (2.90%)	6 (2.02%)
	Pericardial effusion	–	6 (2.90%)	6 (2.02%)
	Ascites	–	3 (1.45%)	3 (1.01%)
	Biopsy specimen	–	2 (0.97%)	2 (0.67%)
	Vaginal secretions	–	1 (0.48%)	1 (0.34%)

PTB, pulmonary tuberculosis; EPTB, extrapulmonary tuberculosis; IGRA, interferon-gamma release assay (IGRA).

### Comparision of Xpert and AutoSAT for the detection of PTB

Based on the CRS as gold standard, Xpert demonstrated significantly higher sensitivity than AutoSAT (30.16% vs. 28.57%, *p* < 0.001) among the 63 confirmed PTB. With bacteriological confirmation as the gold standard, identical sensitivity (94.44%) was observed between Xpert and AutoSAT among the 18 bacteriologically confirmed PTB patients. The higher specificities of AutoSAT than Xpert (100% vs. 96.30%) for MTB detection was observed among PTB, although the difference was not statistically significant (*p =* 0.803). Compared to Xpert, PPV and NPV values were higher in AutoSAT for confirmed PTB (100% vs. 95.00% for PPV; 37.50% vs. 37.14% for NPV) and bacteriologically confirmed PTB (100% vs. 94.44% for PPV; 96.43% vs. 96.30% for NPV), respectively. The agreement between Xpert and AutoSAT was almost perfect among confirmed PTB patients (*κ* = 0.885) (Table 2).

**Table 2:** The accuracy of Xpert and AutoSAT for the diagnosis of PTB.

Group	Sensitivity, % (*n*/*N*)		Specificity, % (*n*/*N*)		PPV		NPV		Xpert/AutoSAT Kappa
	Xpert	AutoSAT	Xpert	AutoSAT	Xpert	AutoSAT	Xpert	AutoSAT	
Confirmed PTB	30.16% (19/63)	28.57% (18/63)	96.30% (26/27)	100% (27/27)	95% (19/20)	100% (18/18)	37.14% (26/70)	37.50% (27/72)	0.885
Bacteriologically confirmed PTB	94.44% (17/18)	94.44% (17/18)			94.44% (17/18)	100% (17/17)	96.30% (26/27)	96.43% (27/28)	-

PTB, pulmonary tuberculosis; PPV, positive predictive value; NPV, negative predictive value.

### Diagnostic performance of the Xpert and AutoSAT in EPTB

The higher sensitivity of AutoSAT than Xpert for MTB detection was observed among confirmed EPTB (22.07% vs. 18.62%, *p* < 0.001) and bacteriologically confirmed EPTB patients (92.31% vs. 84.62%, *p* = 0.53). The identical specificity (100%) and PPV (100%) were observed between Xpert and AutoSAT. The AutoSAT showed higher NPV than Xpert (35.43% vs. 34.44% for confirmed PTB; 96.88% vs. 94.94% for bacteriologically confirmed PTB). The agreement between Xpert and AutoSAT was substantial among confirmed EPTB patients (κ = 0.766) (Table 3).

**Table 3:** The accuracy of Xpert and AutoSAT for the diagnosis of EPTB.

Group	Sensitivity, % (*n*/*N*)		Specificity, % (*n*/*N*)		PPV		NPV		Xpert/AutoSAT Kappa
	Xpert	AutoSAT	Xpert	AutoSAT	Xpert	AutoSAT	Xpert	AutoSAT	
Confirmed EPTB	18.62% (27/145)	22.07% (32/145)	100% (62/62)	100% (62/62)	100% (27/27)	100% (32/32)	34.44% (30/64)	35.43% (27/56)	0.766
Bacteriologically confirmed EPTB	84.62% (22/26)	92.31% (24/26)			100% (22/22)	100% (24/24)	93.94% (62/66)	96.88% (62/64)	–

EPTB, extrapulmonary tuberculosis; PPV, positive predictive value; NPV, negative predictive value.

### Comparision of detection rates among different types of EPTB specimens

Due to the small sample size of ascites, biopsy specimen, and vaginal secretions, we excluded them for further analysis. Considering CRS as the reference, the highest sensitivity of AutoSAT was observed on pus specimen (85.71%), followed by urine (80%). Compared to Xpert, AutoSAT yielded higher sensitivity on pleural fluid (14.91% vs. 9.65%, *p* = 0.276), but significantly lower sensitivity on cerebrospinal fluid (25% vs. 16.67%, *p* = 0.007). The identical sensitivity on pus (85.71%), urine (80%), and pericardial effusion (50%) was observed between Xpert and AutoSAT. Furthermore, the same specificities (100%) were observed in Xpert and AutoSAT assays on different types of specimens. The agreement between Xpert and AutoSAT was substantial (*κ* = 0.750) in cerebrospinal fluid and moderate in pleural fluid specimen (κ = 0.595) (Table 4).

**Table 4:** The accuracy of Xpert and AutoSAT for the discharge diagnosis of EPTB among various specimens.

Specimens	Sensitivity, % (*n*/*N*)		Specificity, % (*n*/*N*)		PPV		NPV		Kappa
	Xpert	AutoSAT	Xpert	AutoSAT	Xpert	AutoSAT	Xpert	AutoSAT	
Pleural fluid	9.65% (11/114)	14.91% (17/114)	100% (46/46)	100% (46/46)	100% (11/11)	100% (17/17)	30.87% (46/149)	32.17% (46/143)	0.595
Pus	85.71% (6/7)	85.71% (6/7)			100% (6/6)	100% (6/6)	85.71% (6/7)	85.71% (6/7)	–
Cerebrospinal fluid	25% (3/12)	16.67% (2/12)			100% (3/3)	100% (2/2)	30.77% (4/13)	28.57% (4/14)	0.75
Urine	80% (4/5)	80% (4/5)			100% (4/4)	100% (4/4)	50% (1/2)	50% (1/2)	–
Pericardial effusion	50% (2/4)	50% (2/4)			100% (2/2)	100% (2/2)	50% (2/4)	50% (2/4)	–

PPV, positive predictive value; NPV, negative predictive value.

### Inconsistent results detected by Xpert and AutoSAT

There were two confirmed PTB cases and three confirmed EPTB cases, who were Xpert positive but AutoSAT negative. And one confirmed PTB case and eight confirmed EPTB cases in pleural fluid specimen were Xpert negative but AutoSAT positive (see Table 5).

**Table 5:** The inconsistent results detected by Xpert and AutoSAT.

Group	Number					
	Confirmed PTB	Bacteriologically confirmed PTB	Confirmed EPTB	Bacteriologically confirmed EPTB	Pleural fluid	Cerebrospinal fluid
Xpert+/AutoSAT-	2	1	3	2	2	1
Xpert-/AutoSAT+	1	1	8	4	8	0

## Discussion

A rapid, simple and sensitive laboratory method used to detect MTB is urgently required to guide TB treatment. In this study, using composite reference standard as the gold standard, we compared the diagnostic utility of the AutoSAT test for detecting MTB in suspected PTB and EPTB patients to that of the Xpert method. According to previous reports, the overall sensitivity for the diagnosis of PTB by SAT-TB assay ranged from 39.2 to 93% (Cui et al., 2012; Fan et al., 2014; Yan et al., 2017). In this study, though AutoSAT exhibited a lower detection rate than that of Xpert (28.57% vs. 30.16%) for all confirmed PTB patients, the AutoSAT sensitivity for the diagnosis of bacteriologically confirmed PTB was the same to that of Xpert (94.44% vs. 94.44%), indicating that AutoSAT is highly effective in identifying tuberculosis cases confirmed by smear or culture, a critical aspect for ensuring accurate diagnosis. Targeted DNA detection methods were easy to induce cross-contamination and inability to distinguish live from dead bacteria (Greco et al., 2006; Speers, 2006; Guerra et al., 2008). However, previous reports showed that SAT might reduce the risk of laboratory contamination, and had lower false-positive rates due to the detection of RNA, which is relatively more stable than DNA and degrades followed the pathogen die (Yan et al., 2016; Chen et al., 2018). In this study, higher specificity was observed for PTB in AutoSAT than that of Xpert (100% vs. 96.30%), indicating that the false-positive results were minimized, which is crucial for avoiding unnecessary treatment and associated costs. Based on the high PPV (100%) of AutoSAT test for PTB, when a sputum sample is AutoSAT positive, the antiTB treatment is most likely to be started in clinically suspected PTB. And with the high value of NPV (96.43%) in bacteriologically confirmed PTB, MTB infection can be excluded based on the negative AutoSAT result. Furthermore, the almost perfect agreement with the widely utilized Xpert (*κ* = 0.885) demonstrated that AutoSAT is a robust diagnostic method in the detection of PTB.

The diagnosis of EPTB is difficult due to the extensive lesions, paucibacillary nature, and complicated clinical manifestations (Li et al., 2023; Norbis et al., 2014). Previous studies revealed that the sensitivity and specificity of the SAT-TB test for the diagnosis of EPTB were 83.6 and 79.4% when compared to culture methods, 41.6 and 100% when compared with the clinical diagnosis (Li et al., 2020). In this study, a higher sensitivity of AutoSAT compared to Xpert was observed, irrespective of the reference standard used, suggesting that AutoSAT was highly beneficial for paucibacillary EPTB patients. Both AutoSAT and Xpert with a specificity of 100% indicates a high level of accuracy in excluding patients who do not have EPTB. Besides, the agreement between Xpert and AutoSAT was substantial, indicating that AutoSAT is a sensitive performance in the diagnosis of EPTB. Various detection rates of MTB were observed in different types of EPTB specimens by AutoSAT, with the higher sensitivity observed on pus (85.71%) and urine specimen (80%), demonstrating superiority for AutoSAT in diagnosis of these specimens from suspected EPTB. The AutoSAT assay outperformed Xpert (14.91% vs. 9.65%) in pleural fluid specimen, providing a valuable diagnostic tool for identifying tuberculous pleurisy (TP) cases.

The inconsistent results between Xpert and AutoSAT mainly occurred in TP cases, with eight Xpert negative but AutoSAT positive, further illustrating that AutoSAT was a more sensitive method for TP. However, in the present study, SAT yielded false-negative results in three EPTB and two PTB patients, which probably due to the treatment history, the efficiency of sample preparation, extraction methods, presence of inhibitors of enzymatic amplification, or uneven distribution of MTB in the samples (Jonas et al., 1993; Reischl et al., 1998).

Several limitations should be acknowledged in this study. First, the sample size was modest, warranting validation through prospective multicenter studies with geographically diverse to establish broader applicability. Second, while the current investigation focused exclusively on adult TB cases, future research should explore the performance of AutoSAT in pediatric populations, where typically lower bacterial loads may present distinct diagnostic challenges. Finally, a rigorous cost-effectiveness analysis will be crucial to assess the feasibility of large-scale implementation, particularly in resource-constrained healthcare settings.

## Conclusion

In conclusion, AutoSAT is an accurate, sensitive and rapid method for the detection of MTB in both PTB and EPTB patients. AutoSAT is highly effective in bacteriologically confirmed PTB, and outperforms Xpert in paucibacillary EPTB patients, especially for pleural fluid specimen detection.

## Data Availability

The original contributions presented in the study are included in the article/supplementary material, further inquiries can be directed to the corresponding authors.
